# Adverse Events Associated with Ethical Kampo Formulations: Analysis of the Domestic Adverse-Event Data Reports of the Ministry of Health, Labor, and Welfare in Japan

**DOI:** 10.1155/2019/1643804

**Published:** 2019-04-15

**Authors:** Yutaka Shimada, Makoto Fujimoto, Tatsuya Nogami, Hidetoshi Watari

**Affiliations:** Department of Japanese Oriental Medicine, Graduate School of Medicine and Pharmaceutical Sciences, University of Toyama, Toyama, Japan

## Abstract

**Objectives:**

Traditional Japanese Kampo medicines have been integrated into the Japanese national health-care system. In Japan, the Ministry of Health, Labor, and Welfare's website discloses adverse drug-event data that have been obtained from medical personnel reports investigated by the Pharmaceutical and Medical Devices Agency. Using these data, we investigated adverse events associated with ethical Kampo formulations.

**Methods:**

Reports of adverse events associated with ethical Kampo formulations from the domestic adverse-event data were obtained from July 30, 2003, to March 31, 2018. Adverse events were then categorized, and the relationships between categories of adverse events and crude drugs were analyzed.

**Results:**

There were 4,232 reported adverse events associated with ethical Kampo formulations. The numbers of events by category were as follows: events related to liver injury, 1,193; lung injury, 1,177; pseudoaldosteronism, 889; mesenteric phlebosclerosis, 223; drug eruption, 185; and others, 565. Among events related to both liver injury and lung injury, approximately 70% were suspected to be induced by Kampo formulations containing Scutellariae Radix. The pseudoaldosteronism-related events, which are induced by Glycyrrhizae Radix, included several events related to muscle injury, heart failure, and arrhythmia. Events related to mesenteric phlebosclerosis, believed to be induced by long-term use of Kampo formulas containing Gardeniae Fructus, increased remarkably during the study period. Among the events related to drug eruption, approximately 35% were suspected to be induced by Kampo formulations containing Ephedrae Herba.

**Conclusion:**

Kampo medicines may cause various adverse events. The present results provide valuable information regarding adverse events associated with Kampo medicines from the viewpoint of patient safety.

## 1. Introduction

Kampo medicine is a traditional Japanese medicine that originated from traditional Chinese medicine and uniquely developed after being introduced into Japan. Currently in Japan, Kampo medicines are available not only as over-the-counter drugs, but also as prescription drugs (i.e., ethical drugs). Indeed, Kampo medicines have been integrated into the national Japanese health-care system. At present, 148 Kampo extract formulations and 187 types of crude drugs have been approved by the Ministry of Health, Labor, and Welfare (MHLW) and are being used under the national health-insurance program [1].

There is no medical license specific to Kampo medicine in Japan, so it is possible for physicians who are not Kampo medicine specialists (not certified by the Japan Society for Oriental Medicine) to prescribe Kampo medicines. The development of modern, ready-to-use forms of Kampo medicines has resulted in increased usage, mainly of spray-dried granular extracts of the original formulas. Kampo extracts for ethical use have largely replaced traditional decoctions of the crude drugs, even though crude drugs are also covered by the national health-insurance system. Japanese physicians with limited knowledge of Kampo medicine tend to prescribe ethical Kampo extracts, rather than crude drugs for decoction, mainly based on their knowledge of conventional Western medicine. The application of ethical Kampo formulations has steadily increased in recent decades, and according to a survey conducted by the Japan Kampo Medicines Manufacturers Association, 89.0% of physicians prescribed Kampo medicines in their daily medical practices in 2011 [2].

It was long thought that Kampo medicines had a mild effect and were rarely associated with adverse events. Historically, reported Kampo medicine-induced adverse events included pseudoaldosteronism caused by Glycyrrhizae Radix (kanzo in Japanese) [3], sympathomimetic effects caused by Ephedrae Herba (mao in Japanese) [4], and aconite intoxication caused by Aconiti Tuber (bushi in Japanese) [5]. However, it has become clear that Kampo medicines can be accompanied by more serious adverse events. In recent decades, it has been reported that Kampo medicines can cause liver [6] and lung injury [7], and an etiological relationship between these injuries and Scutellariae Radix (ogon in Japanese) has been strongly suspected [8, 9]. Furthermore, mesenteric phlebosclerosis has been reported to be associated with long-term use of Kampo formulas containing Gardeniae Fructus (sanshishi in Japanese) [10].

In Japan, all drugs causing adverse events, including suspected causal drugs and drugs for which a causal relationship cannot be ruled out, should be reported to the Pharmaceutical and Medical Devices Agency (PMDA) by pharmaceutical companies, attending physicians, or pharmacists who first identify them [7]. The PMDA was established in 2004 and provides drug-safety information on their website [11]. In addition, the MHLW website has published adverse-event data reports on ethical drugs and over-the-counter drugs since July 30, 2003; these data were obtained from medical personnel reports investigated by the PMDA, which are released approximately every 4 months [12].

Shimodaira et al., using the Japanese Adverse Drug Event Report (JADER) database of PMDA for about 9 years from April 2004 to February 2013, investigated what adverse drug events were likely to occur with Kampo formulations and what Kampo formulations were likely to cause particular adverse events [13]. Although this report is informative, the category of adverse events is based on the classification used in the JADER database itself and did not appropriately indicate the actual situation of adverse events caused by Kampo formulations. In this study, using the adverse-drug-event data reports on the MHLW website for about 15 years, we categorized adverse events more appropriately based on our own judgment and analyzed the current situation of adverse events associated with ethical Kampo formulations in more detail, including categories of adverse events, changes in adverse events over time, and the relationship between event categories and crude drugs.

## 2. Materials and Methods

### 2.1. Materials

This study analyzed the adverse-event data reports on the MHLW website [12]. These reports were obtained from manufacturers, distributors, medical personnel, and others and were investigated by the PMDA. The reports included events with unknown causal relationships to drugs and those for which the relevance to individual drugs had not been evaluated; the reports show the combinations of suspected causal drugs and related adverse events, as well as their numbers. It is possible that a single case could have involved multiple adverse events or multiple suspected causal drugs; a single case could have been reported more than once by multiple companies or due to information updating. Thus, the total of the aggregate calculated values did not indicate the number of actual cases. It is also noteworthy that adverse-event records from April to July 2004 were not disclosed on the MHLW website.

### 2.2. Methods

We identified reported adverse events associated with ethical Kampo formulations using the domestic adverse-event data reports on ethical drugs (July 30, 2003–March 31, 2018) that are reported on the MHLW website. We then categorized events into those related to liver injury, lung injury, pseudoaldosteronism, mesenteric phlebosclerosis, drug eruption, and others and investigated their changes over time. Furthermore, we analyzed the relationships between the categories of adverse events and crude drugs, including Scutellariae Radix, Glycyrrhizae Radix, Gardeniae Fructus, and Ephedrae Herba.

## 3. Results and Discussion

### 3.1. Number of Reported Adverse Events by Category and Changes over Time

**Figure 1:**
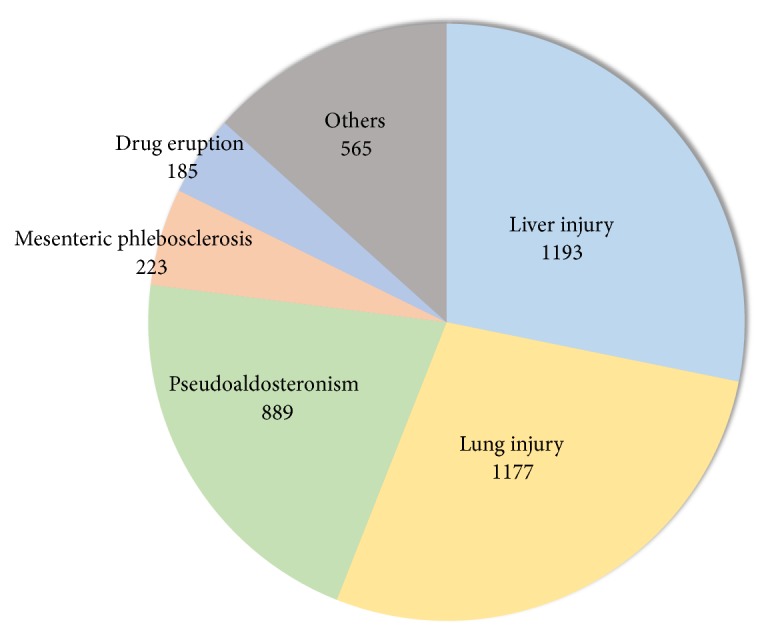
The numbers of reported adverse events associated with ethical Kampo formulations by category.

The total number of reported adverse events in response to ethical Kampo formulations from July 30, 2003, to March 31, 2018, was 4,232. The numbers and percentages of events by category were as follows: events related to liver injury, 1,193 (28.2%); events related to lung injury, 1,177 (27.8%); events related to pseudoaldosteronism, 889 (21.0%); events related to mesenteric phlebosclerosis, 223 (5.3%); events related to drug eruption, 185 (4.4%); and others, 565 (13.3%) (Figure 1). Changes in adverse-event numbers per category over the fiscal years are shown in Table 1. Overall, the numbers of reported adverse events per year tended to increase. In particular, the number of reported adverse events related to mesenteric phlebosclerosis increased remarkably during the study period. The individual reported adverse events related to liver injury, lung injury, pseudoaldosteronism, mesenteric phlebosclerosis, and drug eruption are shown in Tables S1, S2, S3, S4, and S5, respectively.

### 3.2. The Number of Reported Adverse Events Related to Individual Ethical Kampo Formulations

Table 2 shows 148 ethical Kampo formulations listed according to the number of reported adverse events and whether the respective formulations contained Scutellariae Radix, Glycyrrhizae Radix, Gardeniae Fructus, or Ephedrae Herba. In descending order of the number of reported adverse events, these formulations were as follows: shakuyakukanzoto (502), bofutsushosan (321), saireito (305), yokukansan (275), orengedokuto (144), saikokaryukotsuboreito (134), kakkonto (128), hangeshashinto (118), daikenchuto (116), otsujito (113), rikkunshito (107), hochuekkito (106), kamishoyosan (97), shosaikoto (93), bakumondoto (77), seishinrenshiin (77), shin'iseihaito (75), saibokuto (69), saikokeishikankyoto (68), and shoseiryuto (65). A list of ethical Kampo formulations in Japanese and Chinese is shown in Table S6.

The number of reported adverse events related to the top five Kampo formulations and the changes over time are shown in Table 3. In particular, the number of reported adverse events of yokukansan increased remarkably during the study period.

### 3.3. Adverse Events Related to Liver Injury

**Figure 2:**
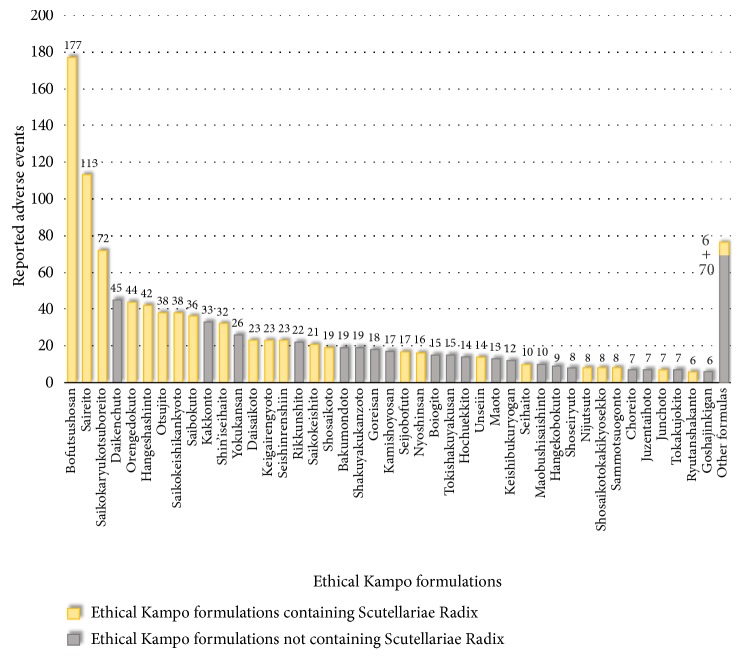
Ethical Kampo formulations suspected of being related to liver injury.

Reported adverse events related to liver injury included liver-function abnormality (467), liver injury (325), drug-induced liver injury (205), jaundice (65), acute hepatitis (57), hepatitis (23), and fulminant hepatitis (15) (Table S1). The ethical Kampo formulations suspected of being related to liver injury are shown in Figure 2 and Table S7; in the order of the number of reported events, these included bofutsushosan (177), saireito (113), saikokaryukotsuboreito (72), daikenchuto (45), orengedokuto (44), hangeshashinto (42), otsujito (38), saikokeishikankyoto (38), saibokuto (36), and kakkonto (33). Eight of these top-ten formulations (all but daikenchuto and kakkonto) contain Scutellariae Radix. Of the total 1,193 reported events related to liver injury, 801 (67.1%) events were suspected to be induced by Kampo formulas containing Scutellariae Radix.

According to an analysis of 1,676 cases of drug-induced liver injury occurring between 1997 and 2006 conducted by the Japan Society of Hepatology, causal drugs were narrowed down to a single drug in 879 cases; 7.1% of them were Kampo medicines [6]. Furthermore, there have been many case reports of liver injury induced by Kampo formulas that contain Scutellariae Radix [14–18]. We also reported a case of recurrent drug-induced liver injury caused by the incidental readministration of a Kampo formula containing Scutellariae Radix [19]. The challenge test provides a definitive etiologic diagnosis of drug-induced liver injury. There have been cases in which Kampo formulas containing Scutellariae Radix were challenged as the causal drug, and the challenge resulted in repeated liver injury [14, 17]. Generally, however, the challenge test is not recommended, as it is accompanied by risks. Cases in which so-called “unintentional challenge tests” were performed incidentally have also been reported. In such cases, the patient took (or the physician prescribed) a Kampo formula containing Scutellariae Radix without realizing that it might result in a challenge test [14–16, 18, 19]. Therefore, the involvement of an immunoallergic mechanism in liver injury induced by Kampo medicine, at least by Scutellariae Radix, has been suggested, as liver injury recurs upon readministration for a short period. As for the incidence of Kampo-medicine-induced liver injury, one study reported that 13 (1.0%) out of 1,328 patients who received Kampo formulas containing Scutellariae Radix developed liver injury [20].

### 3.4. Adverse Events Related to Lung Injury

**Figure 3:**
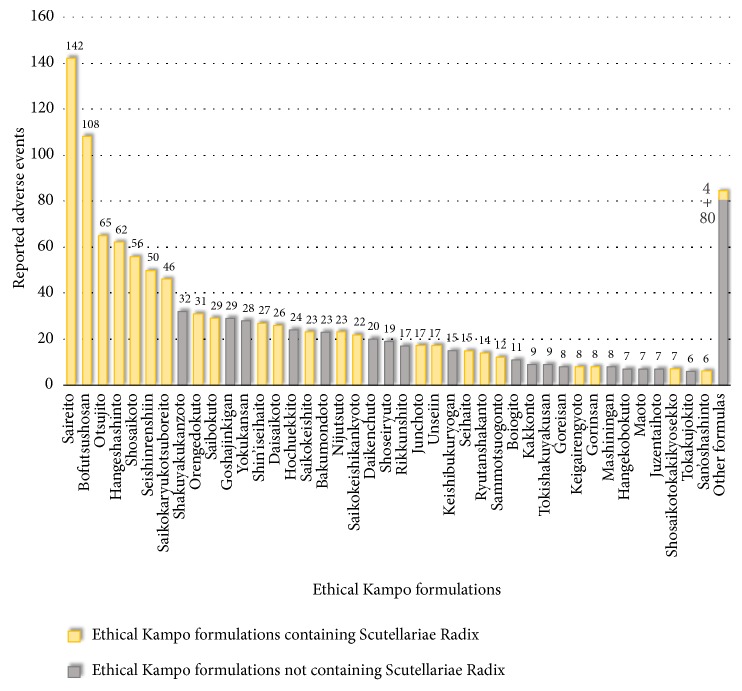
Ethical Kampo formulations suspected of being related to lung injury.

Reported adverse events related to lung injury included interstitial lung disease (852), lung injury (133), pneumonia (114), eosinophilic pneumonia (22), and dyspnea (17) (Table S2). The ethical Kampo formulations suspected of being related to lung injury are shown in Figure 3 and Table S8; in the order of the number of reported events, these include saireito (142), bofutsushosan (108), otsujito (65), hangeshashinto (62), shosaikoto (56), seishinrenshiin (50), saikokaryukotsuboreito (46), shakuyakukanzoto (32), orengedokuto (31), saibokuto (29), and goshajinkigan (29). Nine of these top eleven formulas (all but shakuyakukanzoto and goshajinkigan) contain Scutellariae Radix. Of the total 1,177 reported events related to lung injury, 818 (69.5%) were suspected to be induced by Kampo formulas containing Scutellariae Radix.

In one study, 233 (3.1%) of 7,598 cases of Kampo-medicine-induced interstitial pneumonia were reported from 2004 to 2009 as suspected drug-induced interstitial pulmonary disease [7]. Furthermore, several of the Kampo formulas that induced interstitial pneumonia contain Scutellariae Radix [8, 9]. Indeed, there have been reports of cases in which a Kampo formula containing Scutellariae Radix or Scutellariae Radix alone was challenged, resulting in repeated induction of interstitial pneumonia [21, 22]. Although the causal relationship between Scutellariae Radix and interstitial pneumonia remains unknown, one study reported that peripheral soluble interleukin-2 receptor levels decreased in parallel with an improvement in the clinical course of interstitial pneumonia induced by a Scutellariae Radix-containing formula [23]. This indicates the involvement of an immunoallergic mechanism. In a recent retrospective study using data from a 10-year period, we reported that the incidence of interstitial pneumonia in patients administered Kampo formulas was 0.08% (3/3590), and that in patients administered Kampo formulas containing Scutellariae Radix was 0.27% (3/1111) [24].

### 3.5. Adverse Events Related to Pseudoaldosteronism

Pseudoaldosteronism is induced by Glycyrrhizae Radix [3]. When Glycyrrhizae Radix is ingested orally, glycyrrhizin only slightly penetrates through the gastrointestinal tract epithelium due to its highly hydrophilic sugar moiety. It is absorbed as glycyrrhetinic acid after the sugar moiety is hydrolyzed and converted from glycyrrhizin to glycyrrhetinic acid by enterobacteria in the large intestine [25]. The affinity of cortisol to mineral corticoid receptors is similar to that of aldosterone. Under normal conditions, cortisol is degraded into cortisone in the cytoplasm of renal tubular cells by type 2 11*β*-hydroxysteroid dehydrogenase (11*β*-HSD2). Furthermore, since the affinity of cortisone to mineral corticoid receptors is low, activation of the receptor by cortisol is prohibited. However, in pseudoaldosteronism, it is thought that glycyrrhetinic acid inhibits 11*β*-HSD2 in renal tubular cells, resulting in an accumulation of cortisol that activates mineral corticoid receptors and increases sodium retention and potassium excretion [25–27].

Several investigators have reported that excessive and/or long-term administration of Glycyrrhizae Radix-containing Kampo medicines, crude drug products, or glycyrrhizin frequently leads to pseudoaldosteronism with symptoms such as peripheral edema, hypokalemia, and hypertension [3, 28]. In addition, as a result of pseudoaldosteronism-associated hypokalemia, muscle injury (such as myopathy [29] or rhabdomyolysis [30]), heart injury (such as heart failure [31] or arrhythmia [32, 33]), or glucose intolerance [34] could develop. Furthermore, in pseudoaldosteronism, metabolism of cortisol to cortisone is inhibited, and serum cortisol may be elevated [25]. Therefore, in this study, we categorized reported adverse events related to muscle injury (myopathy, rhabdomyolysis), heart injury (heart failure, arrhythmia), endocrine disorders (blood cortisol increase), and carbohydrate metabolism disorders (hyperglycemia) as adverse events related to pseudoaldosteronism.

**Figure 4:**
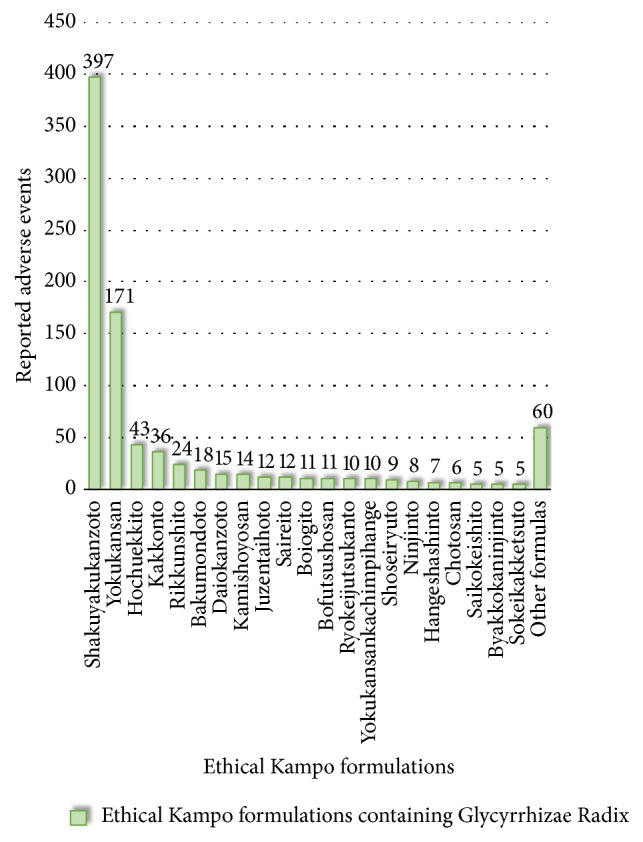
Ethical Kampo formulations suspected of being related to pseudoaldosteronism.

Thus, in total, 889 reported adverse events were categorized as pseudoaldosteronism-related events. Reported adverse events related to pseudoaldosteronism included hypokalemia (291), pseudoaldosteronism (217), rhabdomyolysis (104), myopathy (32), Torsades de pointes (19), heart failure (18), congestive heart failure (16), blood creatine phosphokinase increase (14), ventricular tachycardia (14), ventricular fibrillation (14), peripheral edema (12), and electrocardiogram long QT (12) (Table S3). These were also classified into more detailed categories, including pseudoaldosteronism (217), hypertension-related events (13), edema-related events (25), electrolyte-abnormality-related events (308), muscle-injury-related events (176), heart-failure-related events (46), arrhythmia-related events (95), and others (9) (Table S3). The ethical Kampo formulations suspected of being related to pseudoaldosteronism are shown in Figure 4 and Table S9; in the order of the number of reported events, these were shakuyakukanzoto (397), yokukansan (171), hochuekkito (43), kakkonto (36), rikkunshito (24), bakumondoto (18), daiokanzoto (15), kamishoyosan (14), juzentaihoto (12), and saireito (12).

In Japan, Glycyrrhizae Radix is present in 109 of 148 ethical Kampo extract formulations [25]. Different precautions are described on package inserts of ethical Kampo formulations depending on the amount of Glycyrrhizae Radix they contain (e.g., equal to or more than or less than 2.5 g/day of Glycyrrhizae Radix) [28]. The ethical Kampo extract formulation shakuyakukanzoto contains a large amount of Glycyrrhizae Radix (6 g/day) and is frequently used for the treatment of muscle spasm [35–37]. Yokukansan does not contain as much Glycyrrhizae Radix (1.5 g/day); however, in recent years, it has been widely used in Japan to treat behavioral and psychological symptoms of dementia [38]. Thus, Kampo-formula-associated pseudoaldosteronism development has been considered to be strongly dependent on both the frequency of use and Glycyrrhizae Radix content [39].

### 3.6. Adverse Events Related to Mesenteric Phlebosclerosis

**Figure 5:**
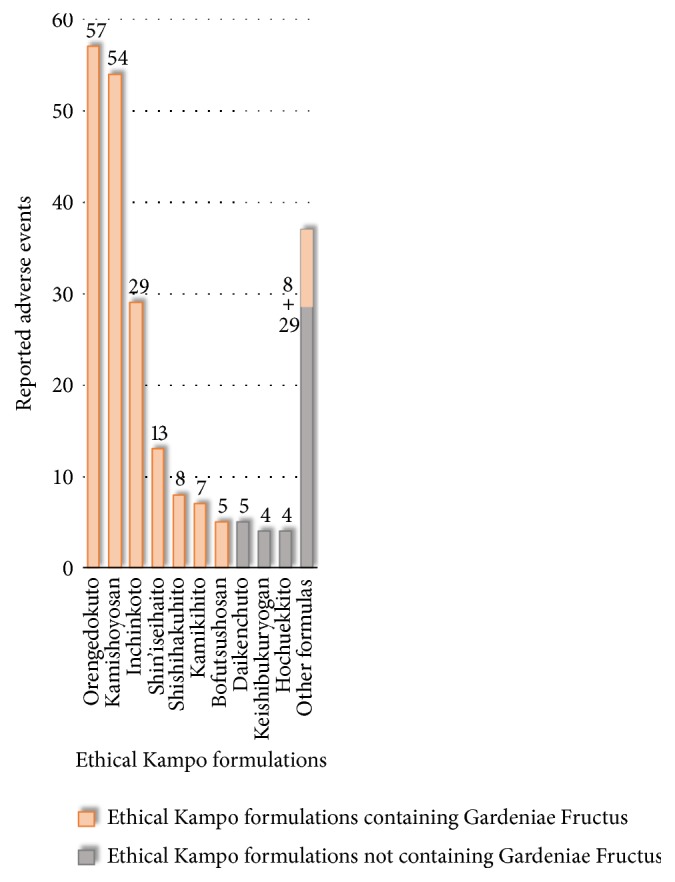
Ethical Kampo formulations suspected of being related to mesenteric phlebosclerosis.

Reported adverse events related to mesenteric phlebosclerosis included mesenteric phlebosclerosis (119), idiopathic mesenteric phlebosclerosis (47), colitis (14), phlebosclerosis (9), ischemic colitis (6), ileus (6), digestive-tract perforation (5), peritonitis (5), intestinal obstruction (3), colon perforation (3), and abdominal pain (3) (Table S4). The ethical Kampo formulations suspected of being related to mesenteric phlebosclerosis are shown in Figure 5 and Table S10; in the order of the number of reported events, these included orengedokuto (57), kamishoyosan (54), inchinkoto (29), shin'iseihaito (13), shishihakuhito (8), kamikihito (7), bofutsushosan (5), daikenchuto (5), keishibukuryogan (4), and hochuekkito (4). Seven of these top-ten formulas (all but daikenchuto, keishibukuryogan, and hochuekkito) contain Gardeniae Fructus. Of all 223 events, 181 (81.2%) were suspected to be induced by Kampo formulas containing Gardeniae Fructus.

Mesenteric phlebosclerosis is a relatively new disease entity [40] also known as phlebosclerotic colitis [41, 42]. Previously, it was often termed idiopathic mesenteric phlebosclerosis [43, 44], as its etiology was unclear. Recently, the number of reports of mesenteric phlebosclerosis involving a history of herbal medicine intake has increased [10, 41–46]. In particular, Gardeniae Fructus is attracting attention as a possible cause, and many of the reported herbal-medicine-related mesenteric phlebosclerosis cases involved a long period of Gardeniae Fructus administration [10, 41, 42, 45, 46].

In Japan, precautions concerning mesenteric phlebosclerosis on the package inserts of orengedokuto, kamishoyosan, and shin'iseihaito were implemented in 2013. Precautions concerning mesenteric phlebosclerosis were also provided for inchinkoto in 2014, and for all ethical Kampo formulas containing Gardeniae Fructus and Gardeniae Fructus as crude drugs in 2018. The precautions were described as follows: As important fundamental caution, “Prolonged administration (usually over 5 years) of a preparation containing Gardeniae Fructus may cause mesenteric phlebosclerosis accompanied by abnormal color tones, edema, erosion, ulcer, and stenosis in the colon. In the case of long-term administration, it is desirable to conduct periodic examinations of computed tomography (CT), colonoscopy, etc.” Furthermore, regarding adverse events, “long-term administration may cause mesenteric phlebosclerosis. If abdominal pain, diarrhea, constipation, feeling of abdominal distension, etc., appear repeatedly, or when fecal occult blood becomes positive, administration should be discontinued, and examinations of CT, large colonoscopy, etc., and appropriate treatment should be carried out. There are also reports of intestinal resection surgery cases.”

In typical mesenteric phlebosclerosis cases, abdominal X-rays and CT scans demonstrate spotted or linear calcification, particularly around the right hemicolon. Bluish-black, bluish-gray, dark purple, or bronze colorations of the colonic membrane are characteristic findings of this disease. In advanced mesenteric phlebosclerosis cases, edema, ulceration, rigidity, and stenosis are also observed by endoscopy [10, 40–46]. Several mesenteric phlebosclerosis cases have also reportedly led to chronic ischemic colitis [40], and multiple cases of colon cancer have coincided with mesenteric phlebosclerosis [47–50]. However, the etiological relationship between colon cancer and mesenteric phlebosclerosis remains unknown, and it is still considered an incidental concurrence. Therefore, we did not include colon cancer as an adverse event related to mesenteric phlebosclerosis in the present study.

### 3.7. Adverse Events Related to Drug Eruption

**Figure 6:**
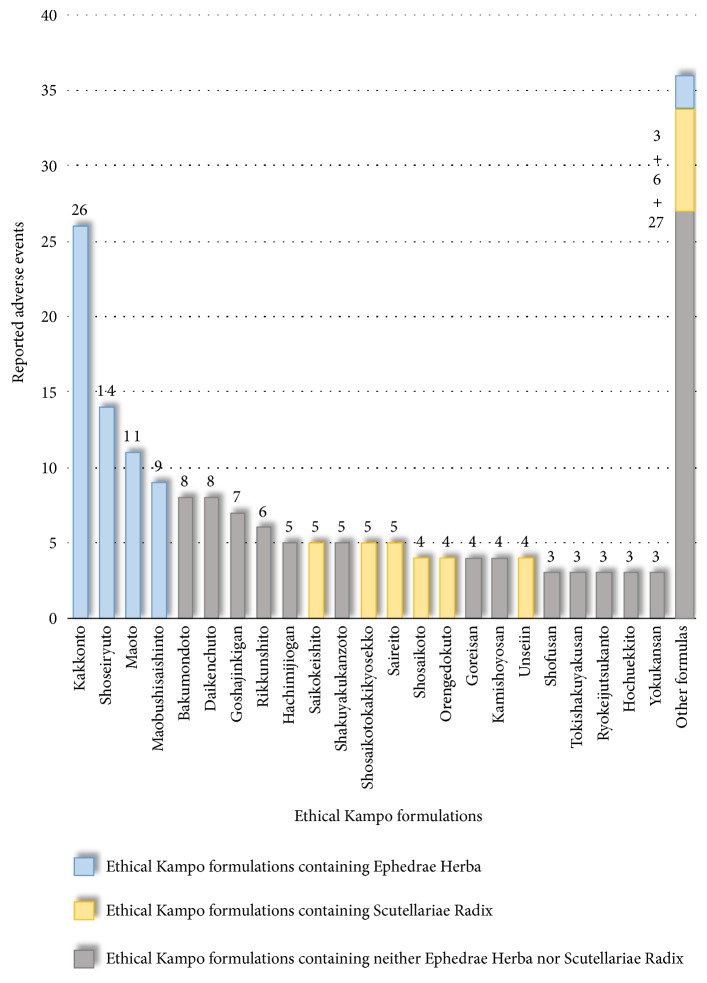
Ethical Kampo formulations suspected of being related to drug eruption.

Reported adverse events related to drug eruption included drug eruption (62), multiple-form erythema (23), Stevens–Johnson syndrome (20), systemic skin eruption (19), and eruption (15) (Table S5). The ethical Kampo formulations suspected of being related to drug eruption are shown in Figure 6 and Table S11; in the order of the number of reported events, these included kakkonto (26), shoseiryuto (14), maoto (11), maobushisaishinto (9), bakumondoto (8), daikenchuto (8), goshajinkigan (7), rikkunshito (6), hachimijiogan (5), saikokeishito (5), shakuyakukanzoto (5), shosaikotokakikyosekko (5), and saireito (5). The top four formulations contain Ephedrae Herba. Of the total 185 reported adverse events, 63 (34.1%) were suspected of being induced by Kampo formulas containing Ephedrae Herba. Several cases of drug eruption are considered to be caused by Ephedrae Herba or ephedrine alkaloid [51–53]. On the other hand, 6 of the top 18 formulas contain Scutellariae Radix, and of the total 185 reported adverse events, 33 (17.8%) were suspected to be induced by Kampo formulas containing Scutellariae Radix.

### 3.8. Other Adverse Events

There were 27 reported adverse events related to anaphylaxis, including anaphylactic shock (14), anaphylaxis reaction (9), and anaphylaxis-like reaction (4). The ethical Kampo formulations suspected of being related to anaphylaxis are shown in Table S12; in the order of the number of reported events, these included kakkonto (3), shoseiryuto (2), kamishoyosan (2), shakuyakukanzoto (2), and shosaikotokakikyosekko (2). Of these 27 reported events, 8 (29.6%) were suspected to be induced by Kampo formulas containing Ephedrae Herba and 6 (22.2%) by Kampo formulas containing Scutellariae Radix.

There were 13 reported adverse events related to allergic cystitis, including cystitis (7), allergic cystitis (4), and eosinophilic cystitis (2). The ethical Kampo formulations suspected of being related to allergic cystitis are shown in Table S13; in the order of the number of reported events, these included shosaikoto (4) and saibokuto (2). Of the total 13 reported adverse events, 12 (92.3%) were suspected to be induced by Kampo formulations containing Scutellariae Radix. There have been several reports of drug-induced cystitis considered to be caused by Kampo formulas containing Scutellariae Radix [54–56].

There were 6 reported adverse events considered to be caused by a sympathomimetic effect of Ephedrae Herba, including urinary retention (4), heart-rate increase (1), and hyperhidrosis (1). The ethical Kampo formulations suspected of being related to a sympathomimetic effect of Ephedrae Herba included shoseiryuto (3), maobushisaishinto (2), and gokoto (1).

There were 2 reported adverse events considered to be aconite intoxication caused by Aconiti Tuber; these included oral hypesthesia (1) and second-degree atrioventricular block (1). The ethical Kampo formulation suspected of being related to aconitine intoxication was maobushisaishinto (2).

### 3.9. Limitations of the Present Study

The numbers of each reported adverse event and the numbers of adverse events associated with each ethical Kampo formulation shown in this study do not indicate the actual numbers of adverse-event cases. The adverse-event data on the MHLW website investigated by the PMDA included events with an unknown causal relationship to drugs, and its relevance to the individual drugs had not been evaluated. It is also possible that multiple adverse events or multiple suspected causal drugs were reported in a single case and that single cases were reported more than once.

The categorization of reported adverse events in this study was based on our own judgment; this may lead to the possibility of bias.

## 4. Conclusions

We analyzed the Japanese domestic reported adverse events of ethical Kampo formulations that were disclosed on the MHLW website after investigation by the PMDA. We identified adverse events related to liver injury, lung injury, pseudoaldosteronism, mesenteric phlebosclerosis, drug eruption, and others. Approximately 70% of liver-injury-related and lung-injury-related events were suspected to be induced by Kampo formulations containing Scutellariae Radix. The number of mesenteric-phlebosclerosis-related events, which is believed to be induced by long-term use of Kampo formulas containing Gardeniae Fructus, increased remarkably over time. The results of the present study provide valuable information that may improve patient safety.

## Figures and Tables

**Table 1:** The number of reported adverse events associated with ethical Kampo formulations per fiscal year by category.

FY	Liver	Lung	PA	MP	Drug	Others	Total
	injury	injury			eruption		
2003^a^	42	27	13	0	8	31	121
2004^b^	55	25	26	0	8	18	132
2005	81	54	29	0	6	9	179
2006	80	52	58	1	8	15	214
2007	60	42	49	1	10	32	194
2008^c^	61	41	51	2	10	22	187
2009^d^	80	68	58	3	17	28	254
2010	75	65	65	13	15	21	254
2011	88	105	48	11	8	22	282
2012	83	105	56	15	11	39	309
2013	80	82	50	41	11	28	292
2014	72	108	48	27	13	23	291
2015	76	84	89	37	10	22	318
2016	142	169	98	34	23	98	564
2017	118	150	151	38	27	157	641

Total	1,193	1,177	889	223	185	565	4,232

FY: Fiscal year until the end of March of the following year; MP: Mesenteric phlebosclerosis; PA: Pseudoaldosteronism.

^a^Data for approximately 8 months, from July 30, 2003 to March 31, 2004.

^b^Data for 8 months, from August 1, 2004 to March 31, 2005. Data from April 1, 2004 to June 30, 2004 are missing.

^c^Data for 11 months, from April 1, 2008 to February 28, 2009.

^d^Data for 13 months, from March 1, 2009 to March 31, 2010.

**Table 2:** Ethical Kampo formulations in the order of the number of reported adverse events.

RO	Ethical Kampo formulation	SR	GR	GF	EH	NEs
1	Shakuyakukanzoto		+			502
2	Bofutsushosan	+	+	+	+	321
3	Saireito	+	+			305
4	Yokukansan		+			275
5	Orengedokuto	+		+		144
6	Saikokaryukotsuboreito	+				134
7	Kakkonto		+		+	128
8	Hangeshashinto	+	+			118
9	Daikenchuto					116
10	Otsujito	+	+			113
11	Rikkunshito		+			107
12	Hochuekkito		+			106
13	Kamishoyosan		+	+		97
14	Shosaikoto	+	+			93
15	Bakumondoto		+			77
15	Seishinrenshiin	+	+			77
17	Shin'iseihaito	+		+		75
18	Saibokuto	+	+			69
19	Saikokeishikankyoto	+	+			68
20	Shoseiryuto		+		+	65
21	Goshajinkigan					63
22	Saikokeishito	+	+			58
23	Daisaikoto	+				52
24	Goreisan					49
25	Boiogito		+			46
26	Maoto		+		+	43
27	Unseiin	+		+		37
27	Maobushisaishinto				+	37
29	Juzentaihoto		+			36
30	Keishibukuryogan					35
30	Keigairengyoto	+	+	+		35
30	Nijutsuto	+	+			35
30	Inchinkoto			+		35
34	Tokishakuyakusan					34
35	Junchoto	+	+			28
36	Seihaito	+	+	+		27
37	Shosaikotokakikyosekko	+	+			26
38	Hangekobokuto					25
39	Tokakujokito		+			23
40	Seijobofuto	+	+	+		22
41	Ryokeijutsukanto		+			21
41	Ryutanshakanto	+	+	+		21
41	Daiokanzoto		+			21
41	Sammotsuogonto	+				21
45	Nyoshinsan	+	+			19
46	Jumihaidokuto		+			18
46	Yokukansankachimpihange		+			18
48	Kamikihito		+	+		14
49	Hachimijiogan					13
49	Keishikajutsubuto		+			13
49	Byakkokaninjinto		+			13
49	Choreito					13
53	Ninjinto		+			12
53	Chotosan		+			12
55	Kakkontokasenkyushin'i		+		+	11
56	Sokeikakketsuto		+			10
56	Gorinsan	+	+	+		10
58	Eppikajutsuto		+		+	9
58	Jidabokuippo		+			9
58	Mashiningan					9
61	Shimbuto					8
61	Makyokansekito		+		+	8
61	Keishikashakuyakuto		+			8
61	Tokiinshi		+			8
61	Ninjin'yoeito		+			8
61	Shishihakuhito		+	+		8
67	Shofusan		+			7
67	Keishikaryukotsuboreito		+			7
67	Tokishigyakukagoshuyushokyoto		+			7
67	Gokoto		+		+	7
67	Bukuryoingohangekobokuto					7
72	Goshuyuto					6
72	Tsudosan		+			6
72	San'oshashinto	+				6
72	Kanzoto		+			6
76	Anchusan		+			5
76	Hangebyakujutsutemmato					5
76	Chikujountanto		+			5
76	Kikyoto		+			5
80	Keishito		+			4
80	Shimotsuto					4
80	Shokenchuto		+			4
80	Daisaikotokyodaio	+				4
80	Keishikaryoujutsubuto		+			4
80	Shakuyakukanzobushito		+			4
86	Shigyakusan		+			3
86	Jizusoippo		+			3
86	Kososan		+			3
86	Nichinto		+			3
86	Ogikenchuto		+			3
86	Keishibukuryogankayokuinin					3
86	Keishikashakuyakudaioto		+			3
93	Goshakusan		+		+	2
93	Kanbakutaisoto		+			2
93	Saikoseikanto	+	+	+		2
93	Daibofuto		+			2
93	Unkeito		+			2
93	Inchingoreisan					2
93	Senkyuchachosan		+			2
93	Keishakuchimoto		+		+	2
93	Kakkonkajutsubuto		+		+	2
93	Ogonto	+	+			2
103	Daiobotanpito					1
103	Kihito		+			1
103	Saikanto	+	+			1
103	Choijokito		+			1
103	Shikunshito		+			1
103	Keishininjinto		+			1
103	Shimpito		+		+	1
103	Jiinshihoto		+			1
103	Choreitogoshimotsuto					1
103	Ryokyojutsukanto		+			1
103	Ryokankyomishingeninto		+			1
103	Hainosankyuto		+			1
103	Seishoekkito		+			1
103	Kyukichoketsuin		+			1
103	Kumibinroto		+			1
103	Kikyosekko					1
103	Keishikaogito		+			1
120	Shohangekabukuryoto					0
120	Mokuboito					0
120	Shichimotsukokato					0
120	Yokuininto		+		+	0
120	Shakanzoto		+			0
120	Jinsoin		+			0
120	Bukuryoin					0
120	Kyukikyogaito		+			0
120	Makyoyokukanto		+		+	0
120	Heiisan		+			0
120	Rokumigan					0
120	Jiinkokato		+			0
120	Shomakakkonto		+			0
120	Tokito		+			0
120	Sansoninto		+			0
120	Rikkosan		+			0
120	Ireito		+			0
120	Orento		+			0
120	Tokikenchuto		+			0
120	Keihito		+			0
120	Daijokito					0
120	Shireito					0
120	Choyoto					0
120	Bushirichuto		+			0
120	Shiunko					0
120	Keishikakakkonto		+			0
120	Keishikakobokukyoninto		+			0
120	Keimakakuhanto		+		+	0
120	Tokishakuyakusankabushi					0

EH: Ephedrae Herba; GF: Gardeniae Fructus; GR: Glycyrrhizae Radix; NEs: Number of reported adverse events; RO: Rank order; SR: Scutellariae Radix.

**Table 3:** The number of reported adverse events for the top five ethical Kampo formulations per fiscal year.

FY	SKT	BTS	SRT	YKS	OGT
2003^a^	11	23	14	1	1
2004^b^	22	11	14	0	1
2005	14	11	15	1	5
2006	38	19	10	3	6
2007	31	27	10	11	6
2008^c^	43	18	12	3	5
2009^d^	27	32	18	15	7
2010	43	16	16	16	6
2011	35	32	13	23	6
2012	47	27	30	19	11
2013	22	21	17	25	32
2014	28	16	27	15	14
2015	33	18	32	24	16
2016	26	26	27	55	15
2017	82	24	50	64	13

Total	502	321	305	275	144

BTS: Bofutsushosan; FY: Fiscal year until the end of March of the following year; OGT: Orengedokuto; SKT: Shakuyakukanzoto; SRT: Saireito; YKS: Yokukansan.

^a^Data for approximately 8 months, from July 30, 2003 to March 31, 2004.

^b^Data for 8 months, from August 1, 2004 to March 31, 2005. Data from April 1, 2004 to June 30, 2004 are missing.

^c^Data for 11 months, from April 1, 2008 to February 28, 2009.

^d^Data for 13 months, from March 1, 2009 to March 31, 2010.

## Data Availability

The data used to support the findings of this study are included within the article and the supplementary information file.

## Abbreviations

11*β*-HSD2: Type 2 11*β*-hydroxysteroid dehydrogenase
JADER: Japanese Adverse Drug Event Report
MHLW: Ministry of Health, Labor, and Welfare
PMDA: Pharmaceutical and Medical Devices Agency.

## Supplementary Materials

Supplementary Materials Table S1: the number of reported adverse events associated with ethical Kampo formulations related to liver injury. Table S2: the number of reported adverse events associated with ethical Kampo formulations related to lung injury. Table S3: the number of reported adverse events associated with ethical Kampo formulations related to pseudoaldosteronism. Table S4: the number of reported adverse events associated with ethical Kampo formulations related to mesenteric phlebosclerosis. Table S5: the number of reported adverse events associated with ethical Kampo formulations related to drug eruption. Table S6: list of ethical Kampo formulations in Japanese and Chinese. Table S7: ethical Kampo formulations suspected of being related to liver injury. Table S8: ethical Kampo formulations suspected of being related to lung injury. Table S9: ethical Kampo formulations suspected of being related to pseudoaldosteronism. Table S10: the ethical Kampo formulations suspected of being related to mesenteric phlebosclerosis. Table S11: ethical Kampo formulations suspected of being related to drug eruption. Table S12: ethical Kampo formulations suspected of being related to anaphylaxis. Table S13: ethical Kampo formulations suspected of being related to allergic cystitis.
