# Supplementary material for: Adverse Events Associated with Ethical Kampo Formulations: Analysis of the Domestic Adverse-Event Data Reports of the Ministry of Health, Labor, and Welfare in Japan
Source: Evid Based Complement Alternat Med. 2019 Apr 15;2019:1643804. doi: 10.1155/2019/1643804 (PMC6500660; doi:10.1155/2019/1643804)
Supplement: Supplementary Materials — Table S1: the number of reported adverse events associated with ethical Kampo formulations related to liver injury. Table S2: the number of reported adverse events associated with ethical Kampo formulations related to lung injury. Table S3: the number of reported adverse events associated with ethical Kampo formulations related to pseudoaldosteronism. Table S4: the number of reported adverse events associated with ethical Kampo formulations related to mesenteric phlebosclerosis. Table S5: the number of reported adverse events associated with ethical Kampo formulations related to drug eruption. Table S6: list of ethical Kampo formulations in Japanese and Chinese. Table S7: ethical Kampo formulations suspected of being related to liver injury. Table S8: ethical Kampo formulations suspected of being related to lung injury. Table S9: ethical Kampo formulations suspected of being related to pseudoaldosteronism. Table S10: the ethical Kampo formulations suspected of being related to mesenteric phlebosclerosis. Table S11: ethical Kampo formulations suspected of being related to drug eruption. Table S12: ethical Kampo formulations suspected of being related to anaphylaxis. Table S13: ethical Kampo formulations suspected of being related to allergic cystitis. [file 1643804.f1.pdf]

**Table S1**. The number of reported adverse events associated with ethical Kampo formulations related to liver injury

| Reported AEs related to liver injury | NEs |
|---|---|
| Drug-induced liver injury | 205 |
| Liver injury | 325 |
| Liver cell damage | 1 |
| Mixed liver damage | 1 |
| Liver function abnormality | 467 |
| Liver function test abnormality | 1 |
| Liver function test value elevation | 3 |
| Liver enzyme elevation | 2 |
| Aspartate aminotransferase increase | 3 |
| Alanine aminotransferase increase | 3 |
| Gamma-glutamyltransferase increase | 3 |
| Blood alkaline phosphatase increase | 1 |
| Hepatitis | 23 |
| Acute hepatitis | 57 |
| Fulminant hepatitis | 15 |
| Chronic hepatitis | 1 |
| Autoimmune hepatitis | 5 |
| Cholestatic hepatitis | 3 |
| Hepatic failure | 3 |
| Acute hepatic failure | 3 |
| Jaundice | 65 |
| Hepatocellular jaundice | 1 |
| Blood bilirubin increase | 1 |
| Hyperbilirubinemia | 1 |
| Total | 1,193 |

AEs: Adverse events; NEs: Number of reported adverse events.

**Table S2**. The number of reported adverse events associated with ethical Kampo formulations related to lung injury

| Reported AEs related to lung injury | NEs |
|---|---|
| Interstitial lung disease | 852 |
| Lung injury | 133 |
| Pneumonia | 114 |
| Eosinophilic pneumonia | 22 |
| Acute eosinophilic pneumonia | 7 |
| Chronic eosinophilic pneumonia | 1 |
| Organizing pneumonia | 5 |
| Idiopathic organizing pneumonia | 1 |
| Cryptogenic organizing pneumonia | 1 |
| Pneumonitis | 8 |
| Allergic alveolitis | 2 |
| Lung fibrosis | 2 |
| Idiopathic interstitial pneumonia | 1 |
| Acute respiratory distress syndrome | 4 |
| Dyspnea | 17 |
| Respiratory failure | 7 |
| Total | 1,177 |

AEs: Adverse events; NEs: Number of reported adverse events.

**Table S3**. The number of reported adverse events associated with ethical Kampo formulations related to pseudoaldosteronism

| Reported AEs related to PA | NEs | |
|---|---|---|
| Pseudoaldosteronism | | 217 |
| Pseudoaldosteronism | 217 | |
| Hypertension-related events | | 13 |
| Hypertension | 7 | |
| Blood pressure elevation | 6 | |
| Edema-related events | | 25 |
| Edema | 7 | |
| Generalized edema | 5 | |
| Peripheral edema | 12 | |
| Body weight increase | 1 | |
| Electrolyte abnormality-related events | | 308 |
| Hypokalemia | 291 | |
| Blood potassium decrease | 10 | |
| Hypernatremia | 2 | |
| Metabolic alkalosis | 5 | |
| Muscle injury-related events | | 176 |
| Myopathy | 32 | |
| Rhabdomyolysis | 104 | |
| Muscle weakness | 6 | |
| Paralysis | 4 | |
| Extremities paralysis | 4 | |
| Extremities paresis | 1 | |
| Asthenia | 6 | |
| Blood creatine phosphokinase increase | 14 | |
| Myalgia | 1 | |
| Tetany | 1 | |
| Myositis | 1 | |
| Muscle enzyme elevation | 1 | |
| Myoglobinuria | 1 | |
| Heart failure-related events | | 46 |
| Heart failure | 18 | |
| Congestive heart failure | 16 | |
| Acute heart failure | 4 | |
| Left ventricular failure | 2 | |
| Right ventricular failure | 1 | |
| Lung edema | 2 | |
| Acute lung edema | 2 | |
| Pericardial effusion | 1 | |
| Arrhythmia-related events | | 95 |
| Arrhythmia | 7 | |
| Supraventricular arrhythmia | 2 | |
| Ventricular arrhythmia | 4 | |
| Tachyarrhythmia | 2 | |
| Extrasystole | 3 | |
| Supraventricular extrasystole | 1 | |
| Ventricular extrasystole | 4 | |
| Ventricular tachycardia | 14 | |
| Ventricular fibrillation | 14 | |
| Atrial fibrillation | 3 | |
| Atrioventricular block | 1 | |
| Complete atrioventricular block | 3 | |
| Long QT syndrome | 6 | |
| Electrocardiogram long QT | 12 | |
| Torsades de pointes | 19 | |
| Other events | | 9 |
| Cortisol increase | 1 | |
| Blood cortisol increase | 1 | |
| Renin decrease | 1 | |
| Hyperglycemia | 4 | |
| Diabetes mellitus | 2 | |
| Total | | 889 |

AEs: Adverse events; NEs: Number of reported adverse events; PA, pseudoaldosteronism.

**Table S4**. The number of reported adverse events associated with ethical Kampo formulations related to mesenteric phlebosclerosis

| Reported AEs related to MP | NEs |
|---|---|
| Mesenteric phlebosclerosis | 119 |
| Idiopathic mesenteric phlebosclerosis | 47 |
| Phlebosclerosis | 9 |
| Colitis | 14 |
| Ischemic colitis | 6 |
| Intestinal obstruction | 3 |
| Ileus | 6 |
| Digestive tract perforation | 5 |
| Colon perforation | 3 |
| Peritonitis | 5 |
| Colon ulcer | 1 |
| Colon stenosis | 1 |
| Abdominal pain | 3 |
| Lower abdominal pain | 1 |
| Total | 223 |

AEs: Adverse events; MP: Mesenteric phlebosclerosis; NRs: Number of reported adverse events.

**Table S5**. The number of reported adverse events associated with ethical Kampo formulations related to drug eruption

| Reported AEs related to drug eruption | NEs |
|---|---|
| Drug eruption | 62 |
| Drug eruption with eosinophilia and systemic symptom | 3 |
| Drug-induced dermatitis | 5 |
| Eruption | 15 |
| Urticaria | 4 |
| Eczema | 2 |
| Papula | 1 |
| Systemic skin eruption | 19 |
| Pustular skin eruption | 1 |
| Erythema | 3 |
| Multiple form erythema | 23 |
| Systemic erythema | 4 |
| Stevens-Johnson syndrome | 20 |
| Mucocutaneous ocular syndrome | 2 |
| Toxic epidermal necrolysis | 4 |
| Acute generalized exanthematous pustulosis | 6 |
| Exfoliative dermatitis | 2 |
| Dermatitis | 1 |
| Atopic dermatitis | 2 |
| Contact dermatitis | 1 |
| Psoriasis-like dermatitis | 1 |
| Pustular acne | 1 |
| Pemphigoid | 1 |
| Pustular psoriasis | 1 |
| Blister | 1 |
| Total | 185 |

AEs: Adverse events; NEs: Number of reported adverse events.

**Table S6**. List of ethical Kampo formulations in Japanese and Chinese

| Ethical Kampo formulations | |
|---|---|
| In Japanese | In Chinese |
| Anchusan | An Zhong San |
| Bakumondoto | Mai Men Dong Tang |
| Bofutsushosan | Fang Feng Tong Sheng San |
| Boiogito | Fang Yi Huang Qi Tang |
| Bukuryoin | Fu Ling Yin |
| Bukuryoingohangekobokuto | Fu Ling Yin He Ban Xia Hou Pu Tang |
| Bushirichuto | Fu Zi Li Zhong Tang |
| Byakkokaninjinto | Bai Hu Jia Ren Shen Tang |
| Chikujountanto | Zhu Ru Wen Dan Tang |
| Choijokito | Diao Wei Cheng Qi Tang |
| Choreito | Zhu Ling Tang |
| Choreitogoshimotsuto | Zhu Ling Tang He Si Wu Tang |
| Chotosan | Diao Teng San |
| Choyoto | Chang Yong Tang |
| Daibofuto | Da Fang Feng Tang |
| Daijokito | Da Cheng Qi Tang |
| Daikenchuto | Da Jian Zhong Tang |
| Daiobotanpito | Da Huang Mu Dan Pi Tang |
| Daiokanzoto | Da Huang Gan Cao Tang |
| Daisaikoto | Da Chai Hu Tang |
| Daisaikotokyodaio | Da Chai Hu Tang Qu Da Huang |
| Eppikajutsuto | Yue Bi Jia Zhu Tang |
| Gokoto | Wu Hu Tang |
| Goreisan | Wu Ling San |
| Gorinsan | Wu Lin San |
| Goshajinkigan | Niu Che Shen Qi Wan |
| Goshakusan | Wu Ji San |
| Goshuyuto | Wu Zhu Yu Tang |
| Hachimijiogan | Ba Wei Di Huang Wan |
| Hainosankyuto | Pai Nong San Ji Tang |
| Hangebyakujutsutemmato | Ban Xia Bai Zhu Tian Ma Tang |
| Hangekobokuto | Ban Xia Hou Pu Tang |
| Hangeshashinto | Ban Xia Xie Xin Tang |
| Heiisan | Ping Wei San |
| Hochuekkito | Bu Zhong Yi Qi Tang |
| Inchingoreisan | Yin Chen Wu Ling San |
| Inchinkoto | Yin Chen Hao Tang |
| Ireito | Wei Ling Tang |
| Jidabokuippo | Zhi Da Pu Yi Fang |
| Jiinkokato | Zi Yin Jiang Huo Tang |
| Jiinshihoto | Zi Yin Zhi Bao Tang |
| Jinsoin | Can Su Yin |
| Jizusoippo | Zhi Tou Chuang Yi Fang |
| Jumihaidokuto | Shi Wei Bai Du Tang |
| Junchoto | Run Chang Tang |
| Juzentaihoto | Shi Quan Da Bu Tang |
| Kakkonkajutsubuto | Ge Gen Jia Zhu Fu Tang |
| Kakkonto | Ge Gen Tang |
| Kakkontokasenkyushin'i | Ge Gen Tang Jia Chuan Xiong Xin Yi |
| Kamikihito | Jia Wei Gui Pi Tang |
| Kamishoyosan | Jia Wei Xiao Yao San |
| Kanbakutaisoto | Gan Mai Da Zao Tang |
| Kanzoto | Gan Cao Tang |
| Keigairengyoto | Jing Jie Lian Qiao Tang |
| Keihito | Qi Pi Tang |
| Keimakakuhanto | Gui Ma Ge Ban Tang |
| Keishakuchimoto | Gui Shao Zhi Mu Tang |
| Keishibukuryogankayokuinin | Gui Zhi Fu Ling Wan Jia Yi Yi Ren |
| Keishibukuryogan | Gui Zhi Fu Ling Wan |
| Keishikajutsubuto | Gui Zhi Jia Zhu Fu Tang |
| Keishikakakkonto | Gui Zhi Jia Ge Gen Tang |
| Keishikakobokukyoninto | Gui Zhi Jia Hou Pu Xing Ren Tang |
| Keishikaogito | Gui Zhi Jia Huang Qi Tang |
| Keishikaryoujutsubuto | Gui Zhi Jia Ling Zhu Fu Tang |
| Keishikaryukotsuboreito | Gui Zhi Jia Long Gu Mu Li Tang |
| Keishikashakuyakudaioto | Gui Zhi Jia Shao Yao Da Huang Tang |
| Keishikashakuyakuto | Gui Zhi Jia Shao Yao Tang |
| Keishininjinto | Gui Zhi Ren Shen Tang |
| Keishito | Gui Zhi Tang |
| Kihito | Gui Pi Tang |
| Kikyosekko | Jie Geng Shi Gao |
| Kikyoto | Jie Geng Tang |
| Kososan | Xiang Su San |
| Kumibinroto | Jiu Wei Bing Lang Tang |
| Kyukichoketsuin | Xiong Gui Diao Xue Yin |
| Kyukikyogaito | Xiong Gui Jiao Ai Tang |
| Makyokansekito | Ma Xing Gan Shi Tang |
| Makyoyokukanto | Ma Xing Yi Gan Tang |
| Maobushisaishinto | Ma Huang Fu Zi Xi Xin Tang |
| Maoto | Ma Huang Tang |
| Mashiningan | Ma Zi Ren Wan |
| Mokuboito | Mu Fang Yi Tang |
| Nichinto | Er Chen Tang |
| Nijutsuto | Er Zhu Tang |
| Ninjin'yoeito | Ren Shen Yang Rong Tang |
| Ninjinto | Ren Shen Tang |
| Nyoshinsan | Nu Shen San |
| Ogikenchuto | Huang Qi Jian Zhong Tang |
| Ogonto | Huang Qin Tang |
| Orengedokuto | Huang Lian Jie Du Tang |
| Orento | Huang Lian Tang |
| Otsujito | Yi Zi Tang |
| Rikkosan | Li Xiao San |
| Rikkunshito | Liu Jun Zi Tang |
| Rokumigan | Liu Wei Wan |
| Ryokankyomishingeninto | Ling Gan Jiang Wei Xin Xia Ren Tang |
| Ryokeijutsukanto | Ling Gui Zhu Gan Tang |
| Ryokyojutsukanto | Ling Jiang Zhu Gan Tang |
| Ryutanshakanto | Long Dan Xie Gan Tang |
| Saibokuto | Chai Pu Tang |
| Saikanto | Chai Xian Tang |
| Saikokaryukotsuboreito | Chai Hu Jia Long Gu Mu Li Tang |
| Saikokeishikankyoto | Chai Hu Gui Zhi Qian Jiang Tang |
| Saikokeishito | Chai Hu Gui Zhi Tang |
| Saikoseikanto | Chai Hu Qing Gan Tang |
| Saireito | Chai Ling Tang |
| Sammotsuogonto | San Wu Huang Qin Tang |
| San'oshashinto | San Huang Xie Xin Tang |
| Sansoninto | Suan Zao Ren Tang |
| Seihaito | Qing Fei Tang |
| Seijobofuto | Qing Shang Fang Feng Tang |
| Seishinrenshiin | Qing Xin Lian Zi Yin |
| Seishoekkito | Qing Shu Yi Qi Tang |
| Senkyuchachosan | Chuan Xiong Cha Diao San |
| Shakanzoto | Zhi Gan Cao Tang |
| Shakuyakukanzobushito | Shao Yao Gan Cao Fu Zi Tang |
| Shakuyakukanzoto | Shao Yao Gan Cao Tang |
| Shichimotsukokato | Qi Wu Jiang Xia Tang |
| Shigyakusan | Si Ni San |
| Shikunshito | Si Jun Zi Tang |
| Shimbuto | Zhen Wu Tang |
| Shimotsuto | Si Wu Tang |
| Shimpito | Shen Mi Tang |
| Shin'iseihaito | Xin Yi Qing Fei Tang |
| Shireito | Si Ling Tang |
| Shishihakuhito | Zhi Zi Bai Pi Tang |
| Shiunko | Zi Yun Gao |
| Shofusan | Xiao Feng San |
| Shohangekabukuryoto | Xiao Ban Xia Jia Fu Ling Tang |
| Shokenchuto | Xiao Jian Zhong Tang |
| Shomakakkonto | Sheng Ma Ge Gen Tang |
| Shosaikoto | Xiao Chai Hu Tang |
| Shosaikotokakikyosekko | Xiao Chai Hu Tang Jia Jie Geng Shi Gao |
| Shoseiryuto | Xiao Qing Long Tang |
| Sokeikakketsuto | Shu Jing Huo Xue Tang |
| Tokakujokito | Tao He Cheng Qi Tang |
| Tokiinshi | Dang Gui Yin Zi |
| Tokikenchuto | Dang Gui Jian Zhong Tang |
| Tokishakuyakusan | Dang Gui Shao Yao San |
| Tokishakuyakusankabushi | Dang Gui Shao Yao San Jia Fu Zi |
| Tokishigyakukagoshuyushokyoto | Dang Gui Si Ni Jia Wu Zhu Yu Sheng Jiang Tang |
| Tokito | Dang Gui Tang |
| Tsudosan | Tong Dao San |
| Unkeito | Wen Jing Tang |
| Unseiin | Wen Qing Yin |
| Yokuininto | Yi Yi Ren Tang |
| Yokukansan | Yi Gan San |
| Yokukansankachimpihange | Yi Gan San Jia Chen Pi Ban Xia |

**Table S7**. Ethical Kampo formulations suspected of being related to liver injury

| RO | Ethical Kampo formulations | SR | NEs | RO | Ethical Kampo formulations | SR | NEs |
|---|---|---|---|---|---|---|---|
| 1 | Bofutsushosan | + | 177 | 42 | Ryutanshakanto | + | 6 |
| 2 | Saireito | + | 113 | 42 | Goshajinkigan | | 6 |
| 3 | Saikokaryukotsuboreito | + | 72 | 44 | Hachimijiogan | | 5 |
| 4 | Daikenchuto | | 45 | 44 | Eppikajutsuto | | 5 |
| 5 | Orengedokuto | + | 44 | 44 | Ryokeijutsukanto | | 5 |
| 6 | Hangeshashinto | + | 42 | 44 | Tsudosan | | 5 |
| 7 | Otsujito | + | 38 | 48 | Goshuyuto | | 4 |
| 7 | Saikokeishikankyoto | + | 38 | 48 | Daisaikotokyodaio | + | 4 |
| 9 | Saibokuto | + | 36 | 50 | Keishikaryukotsuboreito | | 3 |
| 10 | Kakkonto | | 33 | 50 | Yokukansankachimpihange | | 3 |
| 11 | Shin'iseihaito | + | 32 | 50 | Keishibukuryogankayokuinin | | 3 |
| 12 | Yokukansan | | 26 | 50 | Inchinkoto | | 3 |
| 13 | Daisaikoto | + | 23 | 50 | Kamikihito | | 3 |
| 13 | Keigairengyoto | + | 23 | 55 | Byakkokaninjinto | | 2 |
| 13 | Seishinrenshiin | + | 23 | 55 | Tokishigyakukagoshuyushokyoto | | 2 |
| 16 | Rikkunshito | | 22 | 55 | Sokeikakketsuto | | 2 |
| 17 | Saikokeishito | + | 21 | 55 | Keishikashakuyakuto | | 2 |
| 18 | Shosaikoto | + | 19 | 55 | Bukuryoingohangekobokuto | | 2 |
| 18 | Bakumondoto | | 19 | 60 | Kakkontokasenkyushin'i | | 1 |
| 18 | Shakuyakukanzoto | | 19 | 60 | Jumihaidokuto | | 1 |
| 21 | Goreisan | | 18 | 60 | Ninjinto | | 1 |
| 22 | Kamishoyosan | | 17 | 60 | Hangebyakujutsutemmato | | 1 |
| 22 | Seijobofuto | + | 17 | 60 | Makyokansekito | | 1 |
| 24 | Nyoshinsan | + | 16 | 60 | Gorinsan | + | 1 |
| 25 | Boiogito | | 15 | 60 | Jizusoippo | | 1 |
| 25 | Tokishakuyakusan | | 15 | 60 | Goshakusan | | 1 |
| 27 | Hochuekkito | | 14 | 60 | Kososan | | 1 |
| 27 | Unseiin | + | 14 | 60 | Shimotsuto | | 1 |
| 29 | Maoto | | 13 | 60 | Saikanto | + | 1 |
| 30 | Keishibukuryogan | | 12 | 60 | Shikunshito | | 1 |
| 31 | Seihaito | + | 10 | 60 | Daiokanzoto | | 1 |
| 31 | Maobushisaishinto | | 10 | 60 | Tokiinshi | | 1 |
| 33 | Hangekobokuto | | 9 | 60 | Jidabokuippo | | 1 |
| 34 | Shoseiryuto | | 8 | 60 | Chikujountanto | | 1 |
| 34 | Nijutsuto | + | 8 | 60 | Daibofuto | | 1 |
| 34 | Shosaikotokakikyosekko | + | 8 | 60 | Shokenchuto | | 1 |
| 34 | Sammotsuogonto | + | 8 | 60 | Hainosankyuto | | 1 |
| 38 | Choreito | | 7 | 60 | Senkyuchachosan | | 1 |
| 38 | Juzentaihoto | | 7 | 60 | Keishikashakuyakudaioto | | 1 |
| 38 | Junchoto | + | 7 | 60 | Keishakuchimoto | | 1 |
| 38 | Tokakujokito | | 7 | 60 | Keishikaryoujutsubuto | | 1 |

NEs: Number of reported adverse events; RO: Rank order; SR: Scutellariae Radix.

**Table S8**. Ethical Kampo formulations suspected of being related to lung injury

| RO | Ethical Kampo formulations | SR | NEs | RO | Ethical Kampo formulations | SR | NEs |
|---|---|---|---|---|---|---|---|
| 1 | Saireito | + | 142 | 42 | Maobushisaishinto | | 4 |
| 2 | Bofutsushosan | + | 108 | 46 | Shimbuto | | 3 |
| 3 | Otsujito | + | 65 | 46 | Choreito | | 3 |
| 4 | Hangeshashinto | + | 62 | 46 | Sokeikakketsuto | | 3 |
| 5 | Shosaikoto | + | 56 | 46 | Keishikashakuyakuto | | 3 |
| 6 | Seishinrenshiin | + | 50 | 46 | Yokukansankachimpihange | | 3 |
| 7 | Saikokaryukotsuboreito | + | 46 | 46 | Chikujountanto | | 3 |
| 8 | Shakuyakukanzoto | | 32 | 46 | Kamikihito | | 3 |
| 9 | Orengedokuto | + | 31 | 53 | Hachimijiogan | | 2 |
| 10 | Saibokuto | + | 29 | 53 | Shofusan | | 2 |
| 10 | Goshajinkigan | | 29 | 53 | Tokishigyakukagoshuyushokyoto | | 2 |
| 12 | Yokukansan | | 28 | 53 | Ryokeijutsukanto | | 2 |
| 13 | Shin'iseihaito | + | 27 | 53 | Keishito | | 2 |
| 14 | Daisaikoto | + | 26 | 53 | Chotosan | | 2 |
| 15 | Hochuekkito | | 24 | 53 | Makyokansekito | | 2 |
| 16 | Saikokeishito | + | 23 | 53 | Nyoshinsan | + | 2 |
| 16 | Bakumondoto | | 23 | 53 | Tokiinshi | | 2 |
| 16 | Nijutsuto | + | 23 | 53 | Jidabokuippo | | 2 |
| 19 | Saikokeishikankyoto | + | 22 | 53 | Gokoto | | 2 |
| 20 | Daikenchuto | | 20 | 64 | Kamishoyosan | | 1 |
| 21 | Shoseiryuto | | 19 | 64 | Keishikaryukotsuboreito | | 1 |
| 22 | Rikkunshito | | 17 | 64 | Eppikajutsuto | | 1 |
| 22 | Junchoto | + | 17 | 64 | Goshuyuto | | 1 |
| 22 | Unseiin | + | 17 | 64 | Byakkokaninjinto | | 1 |
| 25 | Keishibukuryogan | | 15 | 64 | Hangebyakujutsutemmato | | 1 |
| 25 | Seihaito | + | 15 | 64 | Seijobofuto | + | 1 |
| 27 | Ryutanshakanto | + | 14 | 64 | Kihito | | 1 |
| 28 | Sammotsuogonto | + | 12 | 64 | Shimotsuto | | 1 |
| 29 | Boiogito | | 11 | 64 | Choijokito | | 1 |
| 30 | Kakkonto | | 9 | 64 | Saikoseikanto | + | 1 |
| 30 | Tokishakuyakusan | | 9 | 64 | Daiokanzoto | | 1 |
| 32 | Goreisan | | 8 | 64 | Shimpito | | 1 |
| 32 | Keigairengyoto | + | 8 | 64 | Jiinshihoto | | 1 |
| 32 | Gorinsan | + | 8 | 64 | Shokenchuto | | 1 |
| 32 | Mashiningan | | 8 | 64 | Ryokankyomishingeninto | | 1 |
| 36 | Hangekobokuto | | 7 | 64 | Keishikashakuyakudaioto | | 1 |
| 36 | Maoto | | 7 | 64 | Inchinkoto | | 1 |
| 36 | Juzentaihoto | | 7 | 64 | Seishoekkito | | 1 |
| 36 | Shosaikotokakikyosekko | + | 7 | 64 | Kikyoto | | 1 |
| 40 | Tokakujokito | | 6 | 64 | Kikyosekko | | 1 |
| 40 | San'oshashinto | + | 6 | 64 | Kanzoto | | 1 |
| 42 | Jumihaidokuto | | 4 | 64 | Shakuyakukanzobushito | | 1 |
| 42 | Keishikajutsubuto | | 4 | 64 | Kakkonkajutsubuto | | 1 |
| 42 | Ninjin'yoeito | | 4 | 64 | Keishikaogito | | 1 |

NEs: Number of reported adverse events; RO: Rank order; SR: Scutellariae Radix.

**Table S9**. Ethical Kampo formulations suspected of being related to pseudohyperaldosteronism

| RO | Ethical Kampo formulations | NEs | RO | Ethical Kampo formulations | NEs |
|---|---|---|---|---|---|
| 1 | Shakuyakukanzoto | 397 | 25 | Gokoto | 3 |
| 2 | Yokukansan | 171 | 25 | Kikyoto | 3 |
| 3 | Hochuekkito | 43 | 25 | Keishikaryoujutsubuto | 3 |
| 4 | Kakkonto | 36 | 25 | Shakuyakukanzobushito | 3 |
| 5 | Rikkunshito | 24 | 31 | Kakkontokasenkyushin'i | 2 |
| 6 | Bakumondoto | 18 | 31 | Otsujito | 2 |
| 7 | Daiokanzoto | 15 | 31 | Keishikaryukotsuboreito | 2 |
| 8 | Kamishoyosan | 14 | 31 | Maoto | 2 |
| 9 | Juzentaihoto | 12 | 31 | Shigyakusan | 2 |
| 9 | Saireito | 12 | 31 | Keishikashakuyakuto | 2 |
| 11 | Boiogito | 11 | 31 | Kososan | 2 |
| 11 | Bofutsushosan | 11 | 31 | Saibokuto | 2 |
| 13 | Ryokeijutsukanto | 10 | 31 | Seishinrenshiin | 2 |
| 13 | Yokukansankachimpihange | 10 | 40 | Saikokeishikankyoto | 1 |
| 15 | Shoseiryuto | 9 | 40 | Keishito | 1 |
| 16 | Ninjinto | 8 | 40 | Makyokansekito | 1 |
| 17 | Hangeshashinto | 7 | 40 | Tokakujokito | 1 |
| 18 | Chotosan | 6 | 40 | Kanbakutaisoto | 1 |
| 19 | Saikokeishito | 5 | 40 | Tokiinshi | 1 |
| 19 | Byakkokaninjinto | 5 | 40 | Tsudosan | 1 |
| 19 | Sokeikakketsuto | 5 | 40 | Ninjin'yoeito | 1 |
| 22 | Shosaikoto | 4 | 40 | Ryokyojutsukanto | 1 |
| 22 | Keishikajutsubuto | 4 | 40 | Keishikashakuyakudaioto | 1 |
| 22 | Kanzoto | 4 | 40 | Kumibinroto | 1 |
| 25 | Nichinto | 3 | 40 | Kakkonkajutsubuto | 1 |
| 25 | Jidabokuippo | 3 | | | |

NEs: Number of reported adverse events; RO: Rank order.

**Table S10**. The ethical Kampo formulations suspected of being related to mesenteric phlebosclerosis

| RO | Ethical Kampo formulations | GF | NEs |
|---|---|---|---|
| 1 | Orengedokuto | + | 57 |
| 2 | Kamishoyosan | + | 54 |
| 3 | Inchinkoto | + | 29 |
| 4 | Shin'iseihaito | + | 13 |
| 5 | Shishihakuhito | + | 8 |
| 6 | Kamikihito | + | 7 |
| 7 | Bofutsushosan | + | 5 |
| 7 | Daikenchuto | | 5 |
| 9 | Keishibukuryogan | | 4 |
| 9 | Hochuekkito | | 4 |
| 11 | Anchusan | | 3 |
| 11 | Shosaikoto | | 3 |
| 11 | Juzentaihoto | | 3 |
| 11 | Seijobofuto | + | 3 |
| 15 | Hangekobokuto | | 2 |
| 15 | Goreisan | | 2 |
| 15 | Shoseiryuto | | 2 |
| 15 | Unseiin | + | 2 |
| 15 | Shimotsuto | | 2 |
| 15 | Seihaito | + | 2 |
| 21 | Otsujito | | 1 |
| 21 | Saikokaryukotsuboreito | | 1 |
| 21 | Hangeshashinto | | 1 |
| 21 | Shofusan | | 1 |
| 21 | Tokishakuyakusan | | 1 |
| 21 | Daiobotanpito | | 1 |
| 21 | Byakkokaninjinto | | 1 |
| 21 | Gorinsan | + | 1 |
| 21 | Tokakujokito | | 1 |
| 21 | Chikujountanto | | 1 |
| 21 | Ogikenchuto | | 1 |
| 21 | Inchingoreisan | | 1 |
| 21 | Mashiningan | | 1 |

GF: Gardeniae Fructus; NEs: Number of reported adverse events; RO: Rank order.

**Table S11**. Ethical Kampo formulations suspected of being related to drug eruption

| RO | Ethical Kampo formulations | EH | SR | NEs |
|---|---|---|---|---|
| 1 | Kakkonto | + | | 26 |
| 2 | Shoseiryuto | + | | 14 |
| 3 | Maoto | + | | 11 |
| 4 | Maobushisaishinto | + | | 9 |
| 5 | Bakumondoto | | | 8 |
| 5 | Daikenchuto | | | 8 |
| 7 | Goshajinkigan | | | 7 |
| 8 | Rikkunshito | | | 6 |
| 9 | Hachimijiogan | | | 5 |
| 9 | Saikokeishito | | + | 5 |
| 9 | Shakuyakukanzoto | | | 5 |
| 9 | Shosaikotokakikyosekko | | + | 5 |
| 9 | Saireito | | + | 5 |
| 14 | Shosaikoto | | + | 4 |
| 14 | Orengedokuto | | + | 4 |
| 14 | Goreisan | | | 4 |
| 14 | Kamishoyosan | | | 4 |
| 14 | Unseiin | | + | 4 |
| 19 | Shofusan | | | 3 |
| 19 | Tokishakuyakusan | | | 3 |
| 19 | Ryokeijutsukanto | | | 3 |
| 19 | Hochuekkito | | | 3 |
| 19 | Yokukansan | | | 3 |
| 24 | Kakkontokasenkyushin'i | + | | 2 |
| 24 | Otsujito | | + | 2 |
| 24 | Jumihaidokuto | | | 2 |
| 24 | Hangeshashinto | | + | 2 |
| 24 | Boiogito | | | 2 |
| 24 | Juzentaihoto | | | 2 |
| 24 | Tokiinshi | | | 2 |
| 24 | Ninjin'yoeito | | | 2 |
| 32 | Anchusan | | | 1 |
| 32 | Hangekobokuto | | | 1 |
| 32 | Keishikajutsubuto | | | 1 |
| 32 | Keishibukuryogan | | | 1 |
| 32 | Eppikajutsuto | + | | 1 |
| 32 | Shimbuto | | | 1 |
| 32 | Goshuyuto | | | 1 |
| 32 | Ninjinto | | | 1 |
| 32 | Hangebyakujutsutemmato | | | 1 |
| 32 | Chotosan | | | 1 |
| 32 | Keigairengyoto | | + | 1 |
| 32 | Keishikashakuyakuto | | | 1 |
| 32 | Tokakujokito | | | 1 |
| 32 | Jizusoippo | | | 1 |
| 32 | Daibofuto | | | 1 |
| 32 | Seishinrenshiin | | + | 1 |
| 32 | Bukuryoingohangekobokuto | | | 1 |
| 32 | Inchingoreisan | | | 1 |
| 32 | Senkyuchachosan | | | 1 |
| 32 | Kyukichoketsuin | | | 1 |

EH: Ephedrae Herba; NEs: Number of reported adverse events; RO: Rank order; SR: Scutellariae Radix.

**Table S12**. Ethical Kampo formulations suspected of being related to anaphylaxis

| RO | Ethical Kampo formulations | EH | SR | NEs |
|---|---|---|---|---|
| 1 | Kakkonto | + | | 3 |
| 2 | Shoseiryuto | + | | 2 |
| 2 | Kamishoyosan | | | 2 |
| 2 | Shakuyakukanzoto | | | 2 |
| 2 | Shosaikotokakikyosekko | | + | 2 |
| 6 | Otsujito | | + | 1 |
| 6 | Saikokeishikankyoto | | + | 1 |
| 6 | Hangekobokuto | | | 1 |
| 6 | Boiogito | | | 1 |
| 6 | Maoto | + | | 1 |
| 6 | Bakumondoto | | | 1 |
| 6 | Hangebyakujutsutemmato | | | 1 |
| 6 | Choreito | | | 1 |
| 6 | Rikkunshito | | | 1 |
| 6 | Goshakusan | + | | 1 |
| 6 | Jidabokuippo | | | 1 |
| 6 | Shin'iseihaito | | + | 1 |
| 6 | Saireito | | + | 1 |
| 6 | Bukuryoingohangekobokuto | | | 1 |
| 6 | Maobushisaishinto | + | | 1 |
| 6 | Kikyoto | | | 1 |

EH: Ephedrae Herba; NEs: Number of reported adverse events; RO: Rank order; SR: Scutellariae Radix.

**Table S13**. Ethical Kampo formulations suspected of being related to allergic cystitis

| RO | Ethical Kampo formulations | SR | NEs |
|---|---|---|---|
| 1 | Shosaikoto | + | 4 |
| 2 | Saibokuto | + | 2 |
| 3 | Saikokeishito | + | 1 |
| 3 | Seijobofuto | + | 1 |
| 3 | Bofutsushosan | + | 1 |
| 3 | Ryutanshakanto | + | 1 |
| 3 | Saikoseikanto | + | 1 |
| 3 | Shokenchuto | | 1 |
| 3 | Shin'iseihaito | + | 1 |

EH: Ephedrae Herba; NEs: Number of reported adverse events; RO: Rank order; SR: Scutellariae Radix.
